# Coupling between gamma-band power and cerebral blood volume during recurrent acute neocortical seizures

**DOI:** 10.1016/j.neuroimage.2014.04.014

**Published:** 2014-08-15

**Authors:** Sam Harris, Hongtao Ma, Mingrui Zhao, Luke Boorman, Ying Zheng, Aneurin Kennerley, Michael Bruyns-Haylett, Paul G. Overton, Jason Berwick, Theodore H. Schwartz

**Affiliations:** a Department of Psychology, University of Sheffield, Sheffield S10 2TN, UK; b Department of Neurological Surgery, Neurology and Neuroscience, Brain and Mind Research Institute, Brain and Spine Center, Weill Cornell Medical College, New York Presbyterian Hospital, 525 East 68th Street, Box 99, New York, NY 10021, USA; c School of Systems Engineering, University of Reading, Reading RG6 6AH, UK

**Keywords:** Neurovascular coupling, Gamma, 4-AP, Focal epilepsy

## Abstract

Characterization of neural and hemodynamic biomarkers of epileptic activity that can be measured using non-invasive techniques is fundamental to the accurate identification of the epileptogenic zone (EZ) in the clinical setting. Recently, oscillations at gamma-band frequencies and above (> 30 Hz) have been suggested to provide valuable localizing information of the EZ and track cortical activation associated with epileptogenic processes. Although a tight coupling between gamma-band activity and hemodynamic-based signals has been consistently demonstrated in non-pathological conditions, very little is known about whether such a relationship is maintained in epilepsy and the laminar etiology of these signals. Confirmation of this relationship may elucidate the underpinnings of perfusion-based signals in epilepsy and the potential value of localizing the EZ using hemodynamic correlates of pathological rhythms. Here, we use concurrent multi-depth electrophysiology and 2-dimensional optical imaging spectroscopy to examine the coupling between multi-band neural activity and cerebral blood volume (CBV) during recurrent acute focal neocortical seizures in the urethane-anesthetized rat. We show a powerful correlation between gamma-band power (25–90 Hz) and CBV across cortical laminae, in particular layer 5, and a close association between gamma measures and multi-unit activity (MUA). Our findings provide insights into the laminar electrophysiological basis of perfusion-based imaging signals in the epileptic state and may have implications for further research using non-invasive multi-modal techniques to localize epileptogenic tissue.

## Highlights

• We examine multi-band neural-hemodynamic coupling in the 4-AP epilepsy model.
• Ictal gamma-band power is preferentially coupled to cerebral blood volume.
• Ictal gamma-band power is highly correlated to multi-unit activity.

## Introduction

Understanding the effects of epilepsy on the neurovascular unit is fundamental to elucidating the pathophysiology of the disease and for predicting, identifying and localizing epileptic activity. In medically intractable focal epilepsies, the surgical removal of epileptogenic tissue remains the most promising form of treatment. However, successful post-operative outcomes rely on an accurate delineation of the epileptogenic zone (EZ), defined as “the minimum amount of cortex that must be resected (inactivated or completely disconnected) to produce seizure freedom” (Luders et al., 2006). As a result, there has been a great deal of interest in characterizing potential biomarkers of epileptogenic networks, particularly those that may be measured using non-invasive techniques in order for there to be an appreciable clinical application. Recent research, due in part to the advent of powerful digital broad-band electroencephalogram (EEG) systems, has suggested that pathological neural oscillations at gamma-frequencies and above (>~ 30 Hz) are a valuable indicator of epileptogenic tissue in both neocortical and mesiotemporal regions (Andrade-Valenca et al., 2011, Bragin et al., 1999, Jirsch et al., 2006, Medvedev et al., 2011, Worrell et al., 2004, Zijlmans et al., 2012). Furthermore, the clinical amenability of blood-oxygenation level dependent (BOLD) functional magnetic resonance imaging (fMRI) has also led to it being combined with EEG to localize hemodynamic correlates of electrophysiological epileptic events and aid identification of the EZ (Gotman et al., 2006, Salek-Haddadi et al., 2006, Thornton et al., 2010). However, faithful interpretation of fMRI data in terms of underlying neural activation relies on a detailed understanding of neurovascular coupling, which can vary spatially across laminae (Goense et al., 2012) and brain-regions (Devonshire et al., 2012). A typical assumption that is made to facilitate analysis and interpretation of neuroimaging data is that neurovascular coupling is invariant across health and disease. Yet, since pathological brain states such as epilepsy may be associated with altered neurovascular coupling characteristics, the validity of this assumption has been the subject of much investigation with varying methodologies and results (Hamandi et al., 2008, Harris et al., 2013, Ma et al., 2012, Mirsattari et al., 2006, Stefanovic et al., 2005, Voges et al., 2012, Zhao et al., 2009). Further research is therefore needed to elucidate the extent to which neurovascular coupling characteristics are preserved in the epileptic state, in order to improve interpretation of neuroimaging data in the disorder and ensure the legitimacy of routine assumptions which make such techniques more practicable. Under normal conditions, local field potential (LFP) activity, and in particular the gamma-band component of the LFP, is thought to be a more reliable predictor of perfusion-based signals than multi-unit spiking activity (MUA), although the neurophysiological basis for this remains a topic of intense research (Goense and Logothetis, 2008, Logothetis et al., 2001, Niessing et al., 2005, Nir et al., 2007, Sumiyoshi et al., 2012). These reports underscore the potential value for non-invasive perfusion-based neuroimaging studies to probe cognitive processes. However, while there are considerable reports of pathological gamma activity in clinical (Doesburg et al., 2013, Fisher et al., 1992, Herrmann and Demiralp, 2005, Wu et al., 2008) and experimental (Köhling et al., 2000, Medvedev, 2002, Traub et al., 2005) epilepsy, whether pathological gamma activity is preferentially coupled with hemodynamic signals in the epileptic state is untested. Confirmation of this relationship would suggest a common neural driver of perfusion-related signals in health and epilepsy and, since gamma-band neural measures are strongly co-localized to the EZ, highlight the potential for EEG-neuroimaging paradigms to further delineate the EZ through localization of hemodynamic correlates of pathological gamma activity.

With the above in mind, we sought to examine the laminar electrophysiological underpinnings of seizure-related hemodynamic signals during recurrent ictal discharges in the urethane-anesthetized rat using the well-established 4-aminopyridine (4-AP) acute model of focal neocortical epilepsy. This model provides an ideal framework to examine neurovascular coupling in epilepsy, since seizures recur spontaneously and evolve through similar stages as spontaneous events in the human brain (Harris et al., 2013, Ma et al., 2012, Zhao et al., 2009). Using simultaneous high resolution two-dimensional optical imaging spectroscopy (2D-OIS), we show a powerful linear correlation between cerebral blood volume (CBV) and gamma-band power across all cortical laminae, which was most pronounced in layer 5. Furthermore we show that seizure-related gamma-band activity was most closely coupled to multi-unit activity in deeper laminae nearest the presumed EZ. Our findings provide insights into the laminar evolution of neural measures during recurrent seizures and perfusion-based imaging of seizure events for clinical purposes.

## Materials and methods

All procedures described were approved by the UK Home Office under the Animals (Scientific procedures) Act of 1986. Female hooded Lister rats (total *N* = 8 weighing 260–400 g) were kept in a 12-hr dark/light cycle environment at a temperature of 22 °C, with food and water provided ad libitum. The animals were anesthetized with urethane (1.25 g/kg) intraperitoneally, with atropine being administered subcutaneously (0.4 mg/kg) to reduce mucous secretions during surgery. Depth of anesthesia was monitored throughout and supplementary doses of urethane (0.1 ml) were administered if necessary. We chose to use urethane anesthesia (ethyl carbamate) as it preserves excitatory/inhibitory synaptic transmission, unlike many general anesthetics (Sceniak and MacIver, 2006) and provides a persistent and steady depth of surgical anesthesia, reminiscent of natural sleep (Pagliardini et al., 2013). Moreover, neurovascular coupling is preserved under urethane anesthesia, not only insofar that a single whisker deflection elicits a hemodynamic response in the rat somatosensory cortex (Berwick et al., 2008) but also during CO_2_ challenge (Kennerley et al., 2011), which has led to it being a common choice in neuroimaging studies in rat and neurovascular coupling characteristics to be well-documented during both task-related events (e.g. Berwick et al., 2008, Devor et al., 2005, Harris et al., 2013, Huttunen et al., 2008, Kennerley et al., 2011) and resting-state fluctuations (Bruyns‐Haylett et al., 2013). It has also been shown that neither the spatial–temporal pattern of the evoked hemodynamic response (Devor et al., 2005), nor the relationship between neural activity and BOLD fMRI responses (Huttunen et al., 2008), differs between urethane and alpha-chloralose, another anesthetic routinely used in fMRI studies and whose neurovascular coupling characteristics in turn are comparable to a number of other agents (Franceschini et al., 2010).

A homoeothermic blanket (Harvard Apparatus) and rectal probe were used to maintain core body temperature at 37 °C. The animals were tracheotomized to allow artificial ventilation with pressurized room air and monitoring of end-tidal CO_2_. Blood-gas and end-tidal CO_2_ measurements were used to adjust ventilator parameters and maintain the animal within normal physiological limits (average values: pO_2_ = 92 mm Hg ± 9.2, pCO_2_ = 31 mm Hg ± 5.3). The left femoral artery and vein were cannulated to allow the measurement of arterial blood pressure and phenylephrine infusion (0.13 to 0.26 mg/h to maintain normotension between 100 and 110 mm Hg), respectively. The animal was secured in a stereotaxic frame (throughout experimentation), and the skull overlying coordinates 2 mm anterior to lambda to 2 mm anterior of bregma, and from 1 to 6 mm from midline, was thinned to translucency, in order to expose the somatosensory cortex. A circular plastic ‘well’ was located over the cranial window and filled with saline to reduce optical specularities from the brain surface during imaging.

The potassium channel blocker 4-aminopyridine (4-AP, Sigma, 15 mM, 1 μl) was used to elicit focal seizure-like discharges (Ma et al., 2012, Zhao et al., 2009) in the right vibrissal cortex (RVC). After a 30 s baseline recording period, 4-AP was infused at a depth of 1500 μm (i.e. layer 6) via a fluidic port on the multi-channel microelectrode (Neuronexus Technologies, Ann Arbor, MI, USA) over a 5 minute period (0.2 μl/min) using a 10 μl Hamilton syringe and syringe pump (World Precision Instruments Inc., FL, USA). Recordings were made for 50 min following regional injection of 4-AP.

Two-dimensional optical imaging spectroscopy (2D-OIS) was employed to produce 2D images over time of total hemoglobin concentration (Hbt). Under the reasonable assumption of a constant hematocrit, Hbt can be further interpreted as cerebral blood volume (CBV) and will therefore be referred to as the latter in ensuing text (with the exception of when reporting micro-molar concentrations of Hbt). This technique has been described in detail previously (Berwick et al., 2008). Briefly, illumination of the cortex was conducted at four different wavelengths (495 ± 31 nm, 559 ± 16 nm, 575 ± 14 nm and 587 ± 9 nm FWHM) using a Lamda DG-4 high speed filter changer (Sutter Instrument Company, Novata, CA, USA). Image data were recorded using a Dalsa 1M30P camera (Billerica, MA, USA, each pixel representing ~ 75 μm^2^), synchronized to the filter switching (effective frame rate of 8 Hz/wavelength). These were then subjected to spectral analysis consisting of a path length scaling algorithm (PLSA) employing a modified Beer–Lambert law in conjunction with a path-length correction factor for each wavelength used, based on Monte Carlo simulations of light transport through tissue. After each experiment, a ‘dark baseline’ image data-set was obtained, in which the cortex was not illuminated, and later subtracted from 2D-OIS data in order to account for electrical noise arising from the camera system.

**Fig. 1:**
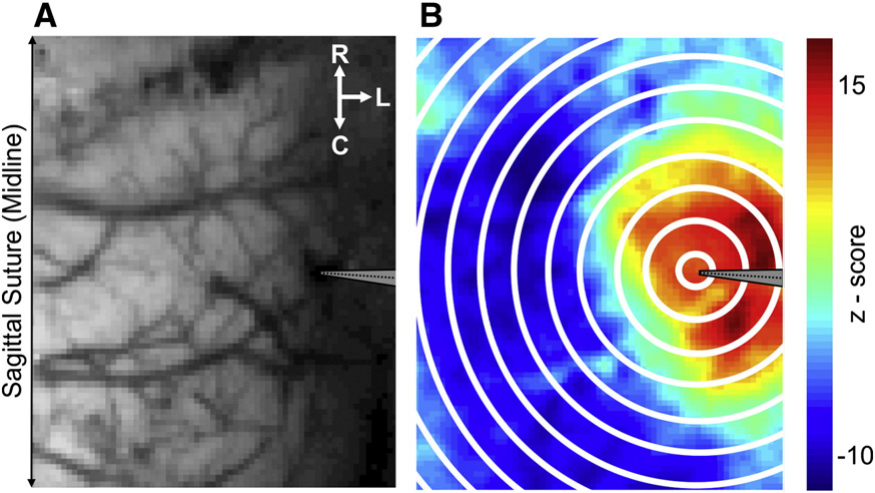
Cortical location of drug-infusion microelectrode and seizure-related SPM analysis. A) Digital photograph of right parietal cortex showing the location of the implanted microelectrode (gray arrow) in the right vibrissal cortex. R = Rostral, L = Lateral and C = Caudal. B) Representative example of ictal SPM analysis, with overlaid concentric rings radiating out from 0.25 mm around the center of microelectrode (gray arrow) in steps of 0.5 mm. Note large *z*-score values (hot colors) within ~ 2.25 mm of focus indicating robust increases in CBV. Conversely, negative *z*-score values (cold colors), indicate decreases in CBV surrounding the focal increase.

In order to localize the region of the somatosensory ‘barrel’ cortex and guide implantation of the multi-channel electrode into the said area, a preparatory 2D-OIS experiment was conducted in each animal. This technique has also been described in detail previously (Berwick et al., 2008). Briefly, the left mystacial pad was electrically stimulated using subcutaneous electrodes (30 trials, 2 s, 5 Hz, 1.2 mA intensity and 0.3 ms pulse width) and recorded image data subjected to the aforementioned spectral analysis. Spatiotemporal changes in Hbt were analyzed using statistical parametric mapping (SPM) in which each pixel's timeseries was regressed against a design matrix representing a direct current (DC) offset, ramp, and ‘boxcar’ function of the same duration as the stimulation. This produced a *z*-score activation map in which pixels within 50% of the maximum *z*-score were used to identify the region contralateral vibrissal cortex activated by somatosensory stimulation. A 16-channel electrode, coupled to a fluidic probe loaded with 4-AP, was inserted into, and normal to, the RVC to a depth of 1500 μm (i.e. layer 6) using a micromanipulator and microscope (example in a representative animal shown in Fig. 1A). Multi-channel electrodes (16 channels with 100 μm spacing, site area 177 μm^2^, 1.5–2.7 MΩ impedance, and 33 μm tip width; Neuronexus Technologies, Ann Arbor, MI, USA) were coupled to a preamplifier and data acquisition device (Medusa BioAmp, TDT, Alachua, FL, USA).

16-channel neural data were recorded continuously throughout each experiment on a floating breadboard fully enclosed by a Faraday cage and sampled at 24 kHz. Raw electrophysiology data were band pass filtered between 0.1 and 300 Hz and down-sampled to 1.2 kHz to yield local field potential (LFP) data. Multi-unit activity (MUA) measures were obtained by band-pass filtering raw electrophysiology data using a 500th order finite impulse response (FIR) filter between 300 and 3000 Hz and full-wave rectification. The threshold for spike detection in each of the 16 channels was calculated as in Quiroga et al., 2004, where *x* is the band-pass filtered signal:

$$4\times \mathrm{median}\left(\frac{\mathrm{abs}\;\left(\;x\;\right)}{0.6745}\right)$$

This method not only accounts for spike classes of either polarity but also minimizes the influence of large amplitude spikes when computing the spike detection threshold (Quiroga et al., 2004). In any pair of consecutive spikes separated by < 1 ms, the spike with the smallest amplitude was disregarded so as to minimize the possibility of detection of false-positives during a spike's refractory period. Finally, a sliding temporal window of 10 ms moving in 1 ms steps was used to determine the spike rate (MUA).

Power spectral density (PSD) measures over time were obtained by applying a Gabor transformation to LFP data. This comprised of a short-term Fourier transform and Gaussian window function of length 500 ms and 250 ms overlap, in the frequency range 0.1–250 Hz. PSD in seven distinct frequency bands were then summated: 0.5–4 Hz (δ-band) 4–7 Hz (θ-band), 7–13 Hz (α-band), 13–25 Hz (β-band), 25–90 Hz (γ-band), 91–150 Hz (High-γ band) and 150–300 Hz (High-Frequency, HF).

Onset and offset times of seizures were computed by first summating PSD in the frequency range 0.1–100 Hz and identifying local maxima corresponding to each seizure using custom-written Matlab^TM^ code. Onset and offset time-points of individual ictal discharges were subsequently defined as 20% of the peak signal power in each seizure epoch. Accurate detection of seizure epochs was confirmed by eye with reference to LFP recordings, with any seizure not beginning or terminating at this signal level omitted from further analysis (e.g. if encroached on by a temporally proximate preceding or ensuing discharge).

Continuous neural measures were then fragmented into individual seizure epochs according to ictal onset and offset timings. Data were summated over each entire seizure epoch in each animal (∑ PSD*_band_*, ∑|LFP| and ∑ MUA) and normalized such that $\bar{\sum {\mathrm{PSD}}_{\mathrm{band}}},\bar{\sum |\mathrm{LFP}|}\;\mathrm{and}\;\bar{\sum \mathrm{MUA}}$ over all detected seizures across all channels was equal to unity. This enabled the aggregation of data across subjects while maintaining information of how neural metrics varied as a function of cortical depth and seizure recurrence. Neural data from microelectrode channels located at depths corresponding to cortical layers 2/3, 4, 5 and 6, were subsequently averaged, according to previously published anatomical data by our laboratory in this species (Devonshire et al., 2007).

To quantify continuous hemodynamic data as a function of distance from the 4-AP infusion site and time, we conducted concentric ring analysis using annuli beginning at 0.25 mm from the injection center and radiating outwardly in steps of 0.5 mm. We chose to disregard the circular area of radius 0.25 mm nearest the center to avoid noise artifacts due to the electrode shank. Continuous hemodynamic time-courses in each concentric ring were normalized to a 30 s pre-infusion baseline which was subsequently set at 104 μmol/L (Kennerley et al., 2009). Continuous hemodynamic data were sub-divided into individual epochs according to the onset and offset times of each seizure. SPM analysis was conducted on each epoch in which the timeseries across each pixel was compared to a design matrix representing a direct current (DC) offset and ‘boxcar’ function of the same duration as the seizure. This produced a *z*-score activation map where positive *z*-scores indicated regional increases in CBV during ictal activity (example of representative ictal SPM map shown in Fig. 1B). The area of CBV activation (CBV_Area_) was calculated from the number of positive pixels in each seizure-SPM map with a *z*-score > 3. For each seizure epoch, hemodynamic time-courses were obtained by averaging the time-series of all pixels within 2.25 mm of the infusion center and normalizing to a 5 s pre-seizure baseline. We then computed the maximum CBV amplitude (CBV_Max_) during the entire epoch. As with neural metrics, all CBV measures were normalized such that $\;\bar{{\mathrm{CBV}}_{\mathrm{Max}}},\mathrm{and}\;\bar{{\mathrm{CBV}}_{\mathrm{Area}}}$ over all detected seizures in each animal was equal to unity.

A particular advantage to our methodology is that physiological noise is robustly minimized. This is because the animal is secured in a passive sphinx-like position by a stereotaxic frame, and the head restrained with ear and bite bars, with the anesthetic regime additionally rendering the animal atonic and nonconvulsive during electrographic seizure activity. Furthermore, our thinned cranial window technique preserves a largely intact central nervous system (with the exception of a small perforation in the dura due to the intra-cranial microelectrode) which, in sum, results in a highly stable preparation. As a result, we, and indeed other laboratories who employ similar methodologies, have not historically found it necessary to account for physiological noise arising from cardiac, respiratory or movement artifacts (Berwick et al., 2008, Bruyns‐Haylett et al., 2013, Harris et al., 2013, Kennerley et al., 2011).

## Results

### Overview of neural-hemodynamic responses following 4-AP seizure induction

**Fig. 2:**
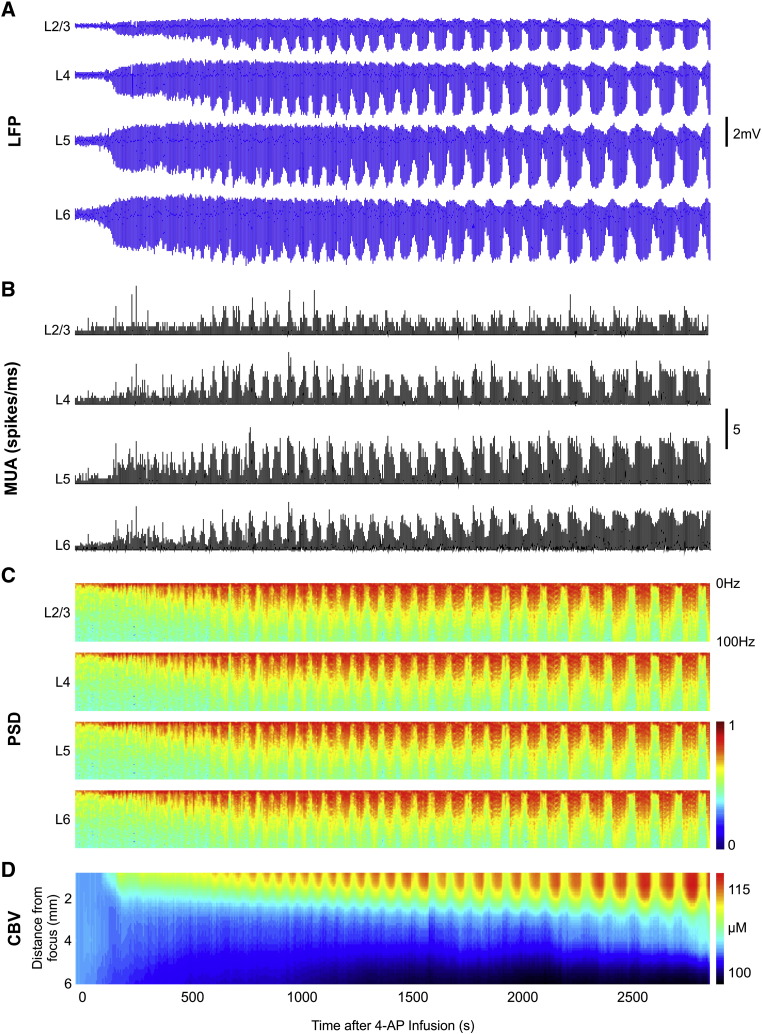
Evolution of neural and hemodynamic responses during and following 4-AP infusion in a representative animal. A) LFP time-courses following 4-AP infusion onset (Time = 0 s) in layers 2/3, 4, 5 and 6. B) Multi-unit activity in layers 2/3, 4, 5 and 6. C) Power spectral density of LFP data shown in A. D) Spatiotemporal analysis of Hbt (CBV) recorded in the same animal.

Infusion of 4-AP into the RVC generated seizure-like discharges as previously described (Ma et al., 2012, Zhao et al., 2009). Shortly after 4-AP infusion onset, pronounced increases in LFP activity were first observed in deeper laminae (associated with the presumed site of the epileptic focus) and subsequently in overlying laminae, suggesting propagation of epileptiform activity from deeper to more superficial cortical depths (representative example of continuous LFP recordings are shown in Fig. 2A). LFP activity consequently evolved into recurrent distinct spontaneous ictal discharges ~ 10 min post-infusion each lasting 50.4 ± 9.1 s (*N* = 180). LFP amplitudes during ictal events appeared to remain approximately constant following seizure induction within each cortical lamina studied, but were comparatively augmented with increasing cortical depth. Similarly, increases in MUA became observable shortly after 4-AP infusion onset and broadly evolved into distinct and progressively more robust seizure-related increases, most prominently in deeper cortical laminae (Fig. 2B). In keeping with the above, spectral power in the 0.1–100 Hz range intensified over time with seizure-related increases becoming progressively more evident (Fig. 2C), particularly at deeper cortical depths. Finally, concurrent CBV (Hbt) measures were also observed to augment over time, with seizure-associated peak concentration and area of activation increasing as ictal discharges recurred (Fig. 2D).

### Multi-band neural activity during recurrent seizure activity

**Fig. 3:**
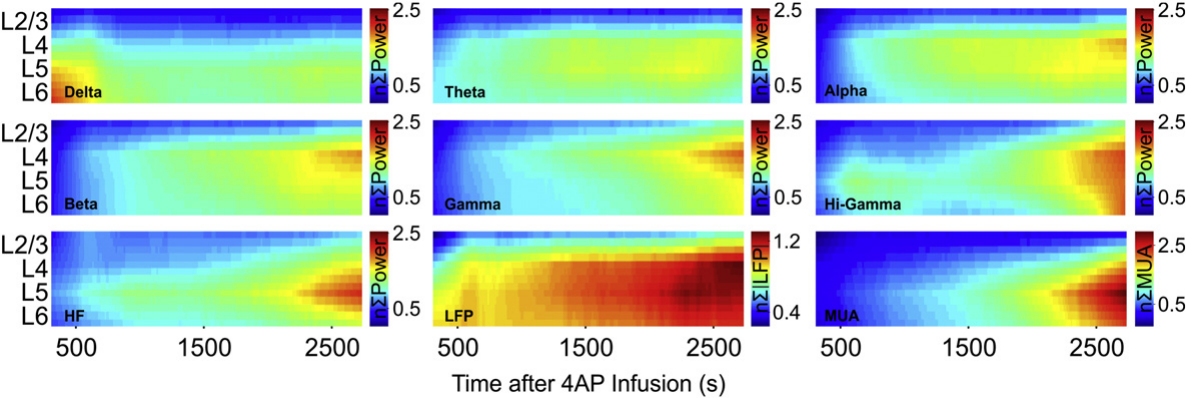
Multi-band power properties during recurrent seizure activity. Spatiotemporal properties of summed delta, theta, alpha, beta, gamma, hi-gamma and high-frequency (HF) band power, and LFP and MUA, as a function of cortical laminae and seizure recurrence (*N* = 180, from 8 subjects).

Normalized multi-band neural data from 180 seizures (8 animals) were collated according to seizure onset time following 4-AP infusion in order to examine changes during recurrent seizure activity. In the main, LFP band measures shared similar dynamics during recurrent seizure activity (Fig. 3). Specifically, seizure related band-power increased shortly after 4-AP infusion onset and progressively intensified during seizure recurrence, predominantly in middle layers and subsequently in underlying laminae, which manifested more strongly with increasing frequency-range. Seizure-related LFP activity exhibited a notable difference in that only modest increases were observed broadly in middle to deeper layers with no clear laminar selectivity (Fig. 3). In contrast, MUA during recurrent seizures initially increased most prominently in deeper layers, with a gradual involvement of overlying laminae (Fig. 3). Generally speaking, neural measures were significantly correlated with seizure onset time across all laminae (Table 1) indicating that recurrent seizure activity was associated with increases in seizure-related multi-band neural activity. A notable exception to this was that of delta-band measures which, contrastingly, exhibited only a significant negative correlation (i.e. decrease) in layer 6 with recurrent seizures. Of the LFP bands studied, gamma-band measures displayed the strongest correlation with seizure onset time across all layers, although MUA was associated with the highest correlations of all multi-band data in layers 5 and 6 (*ρ* = 0.97 and 0.94, respectively, Table 1).

**Table 1:** Coefficients of correlation (Spearman's *ρ*) between each seizure-related multi-band neural measure (∑ PSD*_band_*, ∑|LFP| and ∑ MUA) and associated seizure onset-time following 4AP infusion across cortical laminae (*N* = 180).

	δ-Band	θ-Band	α-Band	β-Band	γ-Band	Hi-γ	HF	LFP	MUA
L2/3	− 0.19	0.26^a^	0.34^a^	0.48^a^	0.63^a^	0.61^a^	0.16^a^	0.39^a^	0.41^a^
L4	0.02	0.55^a^	0.71^a^	0.88^a^	0.92^a^	0.83^a^	0.81^a^	0.74^a^	0.92^a^
L5	0.02	0.59^a^	0.8^a^	0.93^a^	0.93^a^	0.64^a^	0.81^a^	0.74^a^	0.97^a^
L6	− 0.46^a^	0.05a^a^	0.63^a^	0.83^a^	0.84^a^	0.5^a^	0.72^a^	0.43^a^	0.94^a^

a Denotes correlation is significant at the 99% level (i.e. *p* ≤ 0.01, 2-tailed).

Further analysis also revealed that increases in MUA during recurrent seizures were most strongly correlated to gamma-band activity in layers 4, 5 and 6, although robust correlations were observed for the most part in middle to deeper laminae in all but the lowest frequency bands studied (Table 2). Taken together, these observations indicate that recurrent seizures produce intense increases in seizure-related MUA which are in turn more closely allied to increases in gamma-band activity.

**Table 2:** Coefficients of correlation (Spearman's *ρ*) between seizure-related multi-band neural measures (∑ PSD*_band_* and ∑|LFP|) and seizure-related ∑ MUA across cortical laminae (*N* = 180).

	δ-Band	θ-Band	α-Band	β-Band	γ-Band	Hi-γ	HF	LFP
L2/3	0.01	0.19	0.22^a^	0.31^a^	0.53^a^	0.83^a^	0.24^a^	0.35^a^
L4	0.13	0.68^a^	0.78^a^	0.89^a^	0.91^a^	0.83^a^	0.86^a^	0.79^a^
L5	− 0.01	0.6^a^	0.79^a^	0.89^a^	0.9^a^	0.66^a^	0.87^a^	0.75^a^
L6	− 0.34^a^	0.19	0.71^a^	0.89^a^	0.91^a^	0.67^a^	0.87^a^	0.55^a^

a Denotes correlation is significant at the 99% level (i.e. *p* ≤ 0.01, 2-tailed).

### Cerebral blood volume responses during recurrent seizure activity

**Fig. 4:**
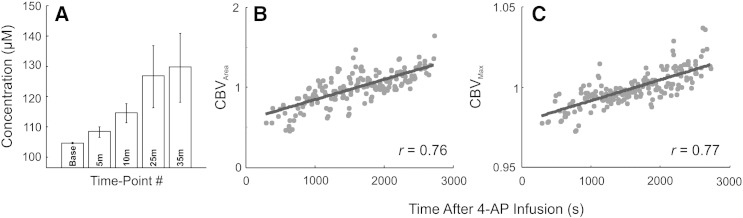
Cerebral blood volume properties during seizure recurrence. A) Averaged Hbt micromolar concentration (i.e. CBV) at five time-points during the recording session (Base = Baseline, 5, 10, 25 and 35 min after infusion onset) indicating a progressive increase over time (*N* = 8). Errors bars are SEM. B) Comparison of seizure-related CBV area (CBV_Area_) and seizure-onset time following 4-AP infusion, indicating a significant linear correlation (Pearson's *r* = 0.76, *p* < 0.001, *N* = 180). C) Comparison of seizure-related peak CBV amplitude (CBV_Max_) and seizure-onset time following 4-AP infusion, also indicating a significant correlation (Pearson's *r* = 0.77, *p* < 0.001, *N* = 180). Linear models fitted using robust least squares linear regression.

We first investigated changes in baseline hemodynamics following 4-AP infusion in each animal, by extracting the average time-course of all pixels within 0.25–2.25 mm of the injection center for the entire recording period and selecting five time-points during the resultant time-series. Firstly, a baseline measure taken 30 s prior to 4-AP infusion, and 5 (i.e. on cessation of 4-AP infusion), 10, 25 and 35 min following infusion onset. This demonstrated an average increase in Hbt concentration from 104.4 ± 0.2 μM to 129.5 ± 11.4 μM (Fig. 4A, *N* = 8). We next compared CBV_Area_ and CBV_Max_ to associated seizure-onset times following 4-AP infusion using seizure-by-seizure analysis (Figs. 4B and C, *N* = 180 discharges from 8 animals). This demonstrated a significant linear relationship between both hemodynamic measures and seizure onset time (Pearson's *r* = 0.76 and 0.77, respectively, *p* < 0.001, in both cases). Taken together, these findings suggest that seizure-related CBV responses are augmented as a function of seizure recurrence which overlie increases in baseline CBV following 4-AP infusion (see also Fig. 2D in a representative animal).

### Neural-hemodynamic coupling during recurrent ictal discharges

**Fig. 5:**
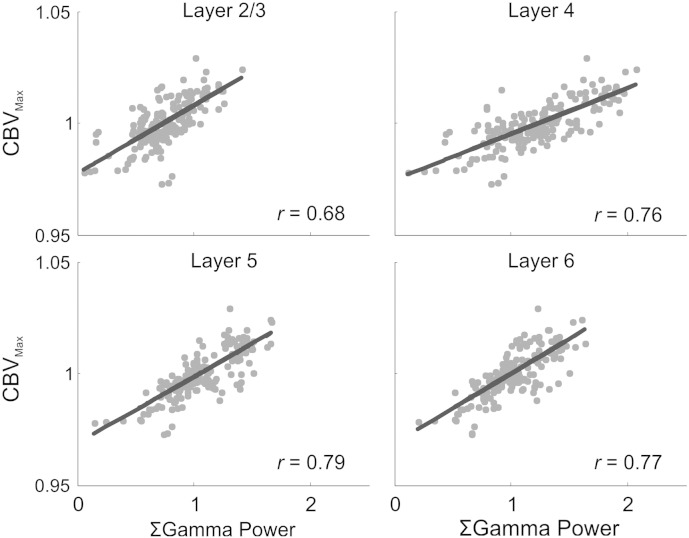
Relationship between seizure-related summed gamma power and peak CBV (CBV_Max_) across laminae. Significant linear correlations between seizure-related summed gamma power and CBV_Max_ in layers 2/3, 4, 5 and 6 (Pearson's *r* = 0.68, 0.76, 0.79 and 0.77, respectively, *p* < 0.01 in all cases, *N* = 178). Linear models fitted using robust (bisquare) linear least squares regression.

In order to identify which of the neural measures examined most faithfully reflected hemodynamic changes, we compared multi-band neural data and peak CBV responses during recurrent seizures. Correlation analysis (Table 3) revealed there to be, in the main, a strong positive relationship between most neural measures and hemodynamics (delta band measures being a notable exception), albeit with the strongest coupling being observed for gamma-band activity across all laminae. Interestingly, seizure-related changes in MUA in middle to deeper layers exhibited the next strongest correlations and were, overall, most closely associated to hemodynamics than LFP measures. Since hemodynamics were most strongly correlated to gamma-band measures, we further examined the nature of the relationship between gamma activity and seizure-related changes in peak CBV (CBV_Max_) responses across laminae (*N* = 178, Fig. 5). This showed there to be a highly significant relationship between gamma-power and CBV_Max_ across all layers which was well described by a linear model (L2/3, Pearson's *r* = 0.68; L4, *r* = 0.76; L5, *r* = 0.79; L6, *r* = 0.77; *p* < 0.01 in all cases, Fig. 5).

**Table 3:** Coefficients of correlation (Spearman's *ρ*) between each seizure-related multi-band neural measure (∑ PSD*_band_*, ∑|LFP| and ∑ MUA) and associated CBV_Max_ (*N* = 178).

	δ-Band	θ-Band	α-Band	β-Band	γ-Band	Hi-γ	HF	LFP	MUA
L2/3	− 0.09	0.25^a^	0.37^a^	0.56^a^	0.66^a^	0.53^a^	0.15	0.39^a^	0.32^a^
L4	0.06	0.46^a^	0.58^a^	0.71^a^	0.74^a^	0.65^a^	0.65^a^	0.6^a^	0.72^a^
L5	0.07	0.35^a^	0.53^a^	0.7^a^	0.77^a^	0.56^a^	0.7^a^	0.52^a^	0.76^a^
L6	− 0.4^a^	− 0.07^a^	0.36^a^	0.65^a^	0.75^a^	0.45^a^	0.64^a^	0.32^a^	0.73^a^

a Denotes correlation is significant at the 99% level (i.e. *p* ≤ 0.01, 2-tailed).

## Discussion

In summary, the key findings described in the current study are the following: although a wide range of multi-band neural measures increased during recurrent seizure activity, MUA increased most strongly, particularly in layer 5. In turn, gamma-band power changes were more closely associated with MUA of all band-limited LFP measures and most strongly correlated with cortical hemodynamic changes in layer 5. These results suggest that gamma-band activity may provide a proxy of population spiking activity during ictal discharges and that hemodynamic correlates of seizure-related gamma-band activity may offer localizing information of epileptiform activity.

Our observation of increased gamma power during seizures in close proximity to the 4-AP infusion site is consistent with previous reports suggesting that increases in gamma activity are highly localized to the seizure onset zone (Andrade-Valenca et al., 2011, Fisher et al., 1992, Medvedev et al., 2011, Worrell et al., 2004). There is considerable evidence for abnormal gamma-band activity in clinical epilepsy syndromes (Doesburg et al., 2013, Fisher et al., 1992, Herrmann and Demiralp, 2005, Wu et al., 2008) and in experimental epilepsy (Köhling et al., 2000, Medvedev, 2002, Traub et al., 2005). Under normal conditions, gamma oscillations are thought to be dependent on fast-spiking parvalbumin-expressing inhibitory interneurons (Cardin et al., 2009). These oscillations have been suggested to ‘bind’ distributed neuronal ensembles into functional networks, thereby playing an important role in information processing and possibly representing a neural correlate of cognition and perception (Engel and Singer, 2001). Conversely, abnormal increases in gamma activity in epilepsy may represent an excessive-binding mechanism, which may underpin sensory hallucinations in complex partial seizures and, through gamma induced changes in synaptic transmission, underlie post-ictal cognitive dysfunction (Medvedev, 2002).

We found LFP sink activity to be distributed approximately equally across depths with little change during recurrent seizures, thus providing little information as to the localization and dynamics of epileptiform activity while, contrastingly, MUA exhibited a preponderance of activity at depths > 400 μm, commensurate with layers 4–6. This spatial discordancy between low-frequency sub-threshold, and high-frequency supra-threshold neural measures, primarily arises due to the latter being under the modulatory influence of feed-forward inhibition. It is therefore an important consideration when using low-frequency neural measures to localize regions of epileptiform activity as these may provide misleading assessment of the epileptogenic zone, compared with the gold-standard metric, namely cells firing bursts of action potentials (Schevon et al., 2012). As conventional EEG measurements in the clinical setting are typically recorded at low-bandwidth (< 100 Hz), identifying a ‘low-frequency’ proxy of population spiking activity during epileptogenesis has received considerable research attention. In this regard, we show gamma-band power to be more closely allied to MUA during ictal discharges, consistent with previous studies showing a correlation between spiking activity and LFP power at gamma frequency ranges under normal conditions (Whittingstall and Logothetis, 2009).

Since the maintenance of elevated neuronal firing rates during gamma oscillations has been recently associated with high oxygen consumption and near-maximal mitochondrial oxidative metabolism (Kann et al., 2011), these observations help to substantiate reports of a close relationship between non-pathological gamma-band activity and perfusion based signals (Goense and Logothetis, 2008, Niessing et al., 2005, Nir et al., 2007, Sumiyoshi et al., 2012). Notwithstanding, reports of coupling between gamma-activity and perfusion-based signals in epilepsy are sparse, although notably a tight correlation between gamma-band activities and cortical glucose metabolism measured by interictal 2-deoxy-2-[^18^F] fluoro-d-glucose (FDG) positron emission tomography (PET) has been demonstrated (Nishida et al., 2008). Indeed, whether, to what extent, and under which conditions, neurovascular coupling characteristics are altered in the epileptic state are topics of ongoing research (Hamandi et al., 2008, Harris et al., 2013, Ma et al., 2012, Mirsattari et al., 2006, Stefanovic et al., 2005, Voges et al., 2012, Zhao et al., 2009) and are important to realizing the diagnostic potential of perfusion-based neuroimaging signals in the disorder. To our knowledge, our study is the first to show a preferential correlation between gamma-band power and cerebral perfusion during recurrent acute focal neocortical seizures. While this study has not explicitly examined whether neurovascular coupling in epilepsy is altered compared to normal conditions, our findings suggest the presence of a common neural driver of perfusion-based signals in normal and epileptic brain states.

Our data also indicate layer 5 to be a key protagonist in the development of epileptiform activity and coupling to hemodynamic signals. This is consistent with previous in-vitro studies showing 4-AP induced epileptiform activity to be linked to excitatory circuits in middle to deep laminae, in particular layer 5, which possesses rich inter- and intra-laminar connectivity and numerous intrinsic bursting neurons (Borbély et al., 2006, Hoffman and Prince, 1995). In addition to being a key site in the initiation of epileptiform discharges, layer 5 has also been implicated to play an important role in the subsequent horizontal spread of epileptiform activity (Telfeian and Connors, 1998). That seizure-related CBV (which is a spatial average over depth) was most strongly correlated to gamma-band activity in layer 5 suggests that perfusion-based signals have the potential to localize the putative major signal source of epileptiform activity. However, it is important to note that significant correlations were observed across all laminae with gamma-band activity not clearly localized to a specific cortical layer, suggesting the possibility of volume conduction effects in which band-limited LFPs spread beyond the primary locus of generation (Kajikawa and Schroeder, 2011). The emergence of high-field human fMRI systems with the ability to examine laminar differences in neurovascular coupling, together with increasingly advanced time-frequency analyses of electrographic data, may further elucidate the laminar nature of gamma-hemodynamic coupling in clinical epilepsy syndromes. This may lead to improved localization of epileptogenic foci using non-invasive multi-modal techniques and guide future ablative therapies involving laminar-specific transections (Nguyen et al., 2011).

A further novel observation was that of the progressive increase in CBV measures during seizure recurrence. Consistent with this, we have previously reported increases in CBV in the epileptogenic focus during singular 4-AP induced ictal discharges, which arise due to robust functional hyperemia associated with seizure-related hypermetabolism (Ma et al., 2012, Zhao et al., 2009). Notwithstanding, if considering the non-linear relationship between CBV and cerebral blood flow (CBF), commonly known as Grubb's power law, our results are at variance with an earlier study demonstrating CBF to be attenuated in later discharges, compared to those occurring earlier, during recurrent seizure activity (Kreisman et al., 1991). Differences in methodology (sodium pentobarbitol anesthesia and *d*-tubocurarine paralysis), epilepsy model (pentylenetetrazol injected intravenously) and inter-seizure interval (of the order of minutes compared to seconds here) may explain the disparity in observations.

An important consideration when interpreting power measures of high-frequency LFP bands is whether spectral energy truly reflects the amount of oscillatory activity within the frequency range of interest or arises due to the methods employed to obtain them. This is particularly true for recordings containing fast transients, for example responses to stimuli and epileptic discharges, whose spectral power are distributed across large frequency ranges (i.e. broadband). Subjecting fast transients to classical time-frequency and filtering methods can therefore result in an output signal with oscillatory behavior despite a non-oscillating input, i.e. a spurious signal with ‘ringing’ artifacts, mathematically known as the Gibbs' phenomenon. Thus, LFPs containing fast transients in the absence of oscillations may exhibit high frequency spectral power leading to an erroneous presumption of high frequency oscillatory activity. It has recently been shown that neuronal spiking is associated with sharp broadband transients in the LFP signal that causes spectral ‘leakage’ into frequencies as low as ~ 50 Hz (i.e. gamma-band), leading to the suggestion that high-frequency activity in LFPs may be a surrogate measure of MUA (Ray and Maunsell, 2011). However, as in a number of previous reports, we have not sought to disassociate the contribution of band-limited oscillatory activity but rather to characterize whether, and to what extent, broadband power changes of LFP signals are related to cortical hemodynamics and track spiking activity during recurrent seizures. Further research is needed to elucidate the functional significance and neurophysiological mechanisms underlying LFP band activity, in particular those at the gamma range and above, given their proposed role in cognitive processes.

In the current study we generated recurrent focal neocortical seizures through local injection of 4-AP in the urethane-anesthetized rat. Though this method remains a model of epilepsy, it has found widespread use in the study of neurovascular coupling in partial onset epilepsy due to it being the only acute model capable of reliably inducing stereotypical focal neocortical ictal-like discharges in the anesthetized rodent (Ma et al., 2012, Zhao et al., 2009). An important caveat, however, is that since this model acutely induces seizure-like discharges in the normal cortex, further research is needed to confirm our findings in the chronic epilepsy condition. We do not consider the possible action of 4-AP on voltage-gated potassium channels expressed on vascular smooth muscle cells to be confounding, since the expected outcome of this would be that of vasoconstriction (and thus a reduction in CBV) in arterioles originating from the middle cerebral artery (Horiuchi et al., 2001).

## Conclusion

In conclusion, we suggest gamma-band activity during ictal discharges to be the most faithful band-limited LFP indicator of epileptogenic activity and most closely associated to cerebral hemodynamics. Our findings may have important implications for the understanding of the electrophysiological basis of seizure-associated hemodynamic responses and be relevant during the localization of epileptogenic foci using multi-modal non-invasive techniques.
